# Challenges to improved therapeutics for metastatic castrate resistant prostate cancer: from recent successes and failures

**DOI:** 10.1186/1756-8722-5-35

**Published:** 2012-07-02

**Authors:** Xuan Huang, Cindy H Chau, William D Figg

**Affiliations:** 1 Medical Oncology Branch, National Cancer Institute, NIH, Building 10, RM 5A01, 9000 Rockville Pike, Bethesda, MD, 20892, USA; 2 Molecular Pharmacology Section, Medical Oncology Branch, National Cancer Institute, NIH, Bethesda, MD, USA

## Abstract

Men with metastatic castration-resistant prostate cancer (mCRPC) carry poor prognosis despite the use of docetaxel-based regimens which has modest survival benefit shown by randomized clinical trials. Significant progress in the discovery of novel therapeutic agents has been made in the past few years. While sipuleucel-T, cabazitaxel, and abiraterone gained regulatory approval in 2010 and 2011, several highly promising candidates/regimens have failed in large scale clinical trials. Challenges remain to optimize the design and interpretation of clinical trial results and develop more effective strategies for mCRPC. In this review, we examined the positive and negative clinical trials in mCRPC in the past and discussed the various aspects of clinical trial design including selection of targets and appropriate outcome measures, biomarker development and implementation, and strategies for combination therapy.

## Introduction

Prostate cancer is the most common non-cutaneous malignancy and the second leading cause of cancer deaths among men in the United States, with an estimated incidence of 241,740 new cases diagnosed and approximately 28,170 men expected to die of the disease in 2012 [1]. Men with metastatic castration-resistant prostate cancer (mCRPC) have a poor prognosis with a median survival of 1–2 years, and until recently, treatment options that improved survival in this setting were limited to docetaxel-based regimens [2,3]. The responses to docetaxel and prednisone are generally short-lived with a modest survival benefit. Recent major advances have resulted in the regulatory approval of sipuleucel-T [4] and cabazitaxel [5] in 2010 and abiraterone acetate (AA) [6] in 2011 for mCRPC patients. Despite these additions to the therapeutic arsenal for this patient population, mCRPC remains incurable and the demand for novel therapies will continue with the pursuit of new druggable targets. In the meantime, we have also witnessed the failure of several highly promising candidates/regimens in late stage of development, reminding us that the road to fighting against prostate cancer is still paved with immense difficulties. Challenges remain for clinical scientists to improve upon existing treatment paradigms and develop more effective strategies for mCRPC. Careful scrutiny of past positive and negative clinical trials will allow us to better optimize target selection, design meaningful outcome measures, and advance biomarker development with implementation in future clinical trials.

## Target selection

Unraveling the molecular make up of cancer cells has resulted in the successful development of specific targeted therapies directed at particular molecular pathways. Studies involving imatinib for CML and GIST, trastuzumab for *HER-2* positive breast cancer, crizotinib for *ALK*-mutated lung cancer, and vemurafenib for *BRAF*-mutated advanced melanoma have all confirmed the significance and feasibility of targeted therapy [7]. Considerable efforts have been made in searching for such targets for prostate cancer patients. Our understanding of the biologic and molecular driving force of prostate cancer growth and progression in the past few years have resulted in investigations of numerous novel targeted therapies, including androgen receptor (AR) targeting agents, tyrosine kinase inhibitors (TKIs), antiangiogenic agents, endothelin receptor antagonists, anti-apoptotic protein inhibitors and proteasome inhibitors [8-10]. Many of them have either received FDA approval or moved to the frontline of late stage development based on improvement of soft intermediate surrogate markers in small phase I/II trials. As bone metastasis is common in patients with mCRPC, agents targeting the bone microenvironment have been successful not only in the prevention of skeletal-related events in men with bone metastases from mCRPC, such as with denosumab and radium-223, but also in improving survival, such as with radium-223 [11,12].

The progression of mCRPC can occur as a result of AR activation despite low levels of androgens. Numerous molecular and genetic aberrations have been postulated to be responsible for gain-of-function changes in the AR signaling pathway under androgen deprivation. This includes intratumoral androgen synthesis, AR protein overexpression, AR gene amplification or point mutations, constitutively active truncated AR splice variants, disturbance of AR-coactivator-corepressor complex, and ligand-independent AR activation by kinase cross-talk [8,13]. Patients who failed primary and/or secondary hormonal therapies are still candidates for more potent novel hormonal agents. AA, a specific inhibitor of the key androgen synthesis enzyme CYP17, and MDV3100, a second generation antiandrogen, represent two such novel therapies targeting the androgen signaling axis in mCRPC. In the recent phase III trial of AA (COU-AA-301) [6], a 35% reduction in the risk of death (HR = 0.65; p < 0.0001) and a 36% increase in median survival (14.8 vs. 10.9 months) were observed in patients treated with AA plus prednisone, compared with patients who received placebo plus prednisone. Encouraging antitumor activity was also recorded in a multicenter phase I/II study of MDV3100 with 56% PSA response and 22% response in soft tissue diseases [14], A randomized, double-blind, multinational phase III trial (AFFIRM), comparing MDV3100 with placebo in mCRPC patients previously treated with docetaxel, was terminated early due to a 4.8 months absolute survival advantage (18.4 vs. 13.6 months) per interim analysis [15].

On the other hand, zibotentan, an endothelin A receptor antagonist blocking endothelin-mediated activation of multiple signaling transduction pathways [16], showed strong inhibition of prostate cancer cell proliferation and delayed progression of bone metastases in preclinical studies [17]. Phase II data demonstrated a statistically significant improvement in overall survival (OS) for patients treated with zibotentan [18]. Interestingly, the study did not meet its primary endpoint of time to progression. Moreover, at the final analysis (median follow-up of 22 months), the absolute OS benefit diminished down to 3.6 months from 7.2 months.[19] Based on the initial OS benefit, an expanded phase III program was launched and included three trials (ENTHUSE M0, ENTHUSE M1, and ENTHUSE M1c) in disease ranging from non-metastatic CRPC (M0) to mCRPC (M1 and M1c), evaluating either single-agent zibotentan (M0 and M1) or in combination with docetaxel (M1c) [20]. However, recent analysis of M1 showed that the study failed to meet its primary endpoint of OS [21]. M0 was also terminated following an early efficacy review indicating that zibotentan monotherapy was unlikely to meet its primary efficacy endpoints. Whether targeting the endothelin axis in prostate cancer is a valid approach remains an open question and results from M1c are eagerly awaited.

Similar disappointing results were reported that led to the termination of the late-stage trial of sunitinib, an antiangiogenic TKI, in combination with prednisone as second-line therapy for mCRPC patients due to the lack of OS prolongation, despite an improvement in progression-free survival (PFS) (5.6 vs. 3.7 months; HR = 0.74; *P* = .0077) [22]. The initiation of this phase III trial was based on the modest anti-tumor activity of sunitinib in two phase II trials [23,24]. One trial showed 6% prostate-specific antigen (PSA) response rate and only one out of 29 evaluable patients manifested as partial response radiographically at week 12 [23]. Some discordance of PSA elevation with radiographic response was observed and considered consistent with PSA dynamics under the unique influence of TKIs, as previously established in a phase II trial of sorafenib in mCRPC [25]. Another phase II trial reported weak antitumor activity with 12.1% PSA response rate and 11.1% radiographic response with single-agent sunitinib [24]. Yet a large phase III trial was implemented since 2008 and has proved to be fruitless. Both examples demonstrate the need to be more selective of agents to bring forward to be tested in the phase III setting.

While recognizing the essential role of early clinical trials in enhancing our understanding of disease biology, we must be conscious about the limitations in interpreting results owing to insufficient sample size, unreliable surrogate markers, or suboptimal outcome measures. Undoubtedly, such trials are required to facilitate efficient screening for active compounds for further clinical development. Selecting appropriate targets for development in phase III trials and the decision to move the drug forward can be difficult at times when early trials reveal only mild clinical activity based on current, suboptimal trial designs. A more stringent and validated benchmark in assessing a meaningful clinical benefit is in enormous demand.

## Appropriate outcome measures

The efficient design of phase II trials to identify active compounds for phase III testing remains a challenge. Alternate end points for screening phase II studies are urgently needed to limit the high incidence of negative phase III trials. Choosing the most suitable primary endpoints for prostate cancer trials has been challenging historically. Although OS is the gold standard for demonstrating clinical benefit due to its objectivity, it requires larger patient numbers and longer follow-up. Survival analysis may also be confounded by crossover or subsequent therapies administered after a study drug is discontinued. Valid intermediate surrogate endpoints have been the center of debate for the past few years. Analyses of PSA response rates, objective response rates and PFS have all been proposed as means to accelerate the drug development process.

Most phase II trials have used PSA decline of more than 50 percent as the primary endpoint, based on its high predictive value of survival from a multivariate analysis [26] and the prostate cancer clinical trials working group’s (PCWG1) recommendation [27]. Some trials also incorporate 3-month 30% PSA decline since it was found to be the optimal biochemical surrogate from a retrospective analysis of the phase III SWOG 9916 trial [28]. However, the limitations of PSA and PSA-based surrogates in predicting survival have been increasingly recognized. In TAX 327, despite a statistical significant improvement in the rates of PSA response in patients taking docetaxel weekly compared to mitoxantrone (48% vs. 32%, p < 0.001, respectively), there was no difference in OS between the two groups [3]. Prospective trials are required to validate PSA-based markers in selected patient populations before they can be confidently used as predictors of survival and as screening tools in early phase trials. Most phase II trials define disease progression by either radiographic or PSA progression as defined by PCWG1 and PCWG2 [27,29]. For consistency of reporting clinical trials, the PCWG2 defined PSA progression as the date that an increase of 25% or more and absolute increase of 2 ng/mL or more from the nadir are documented. For patients who have an initial PSA decline during treatment, this must be confirmed by a second value 3 or more weeks later. The significance of solely using PSA progression without evidence of radiographic or symptomatic progression became questionable as we gained more insight into the mechanisms of action of phase II agents and the natural history of this disease. Several trials involving TKIs have observed rising PSAs after treatment [23,25]. The discordance between the PSA increase and radiographic improvement may be due to the effect of noncytotoxic agents modulating PSA secretion independent of its activity on tumor suppression [30]. Without other well-defined indicators for disease progression, PSA progression is no longer considered representative of treatment failure [29], thus, allowing these agents to be fully evaluated in patients to achieve the maximum possible benefit. As such, the interpretation of post-therapy PSA changes as an outcome measure in the era of targeted agents is of unclear significance [31].

Response rates have also been assessed in patients with measureable diseases on CT scans. However, the clinical picture of mCRPC is dominated by bone metastases in 90-95% of the patients. Radionuclide bone scan is routinely used to assess these bony lesions but the criteria for response categorization are not well-defined and reported in clinical trials. Other imaging modalities (MRI and PET) to assess bone metastasis are still under investigation [32]. Moreover, as cytostatic biologic agents are increasingly being evaluated in clinical trials, the expectation of tumor shrinkage by these agents seems unrealistic. In a small number of patients, usually less than half of the study accrual [33], with soft tissue or visceral metastases that are evaluable, the response rate defined by RECIST criteria is far from being acceptable for cytostatic agents. In the phase II sunitinib and sorafenib trials, the response rates were in 2/18 and 1/23 (combined stage I and II) patients, respectively [24,25,34]. Continuous application of this intermediate surrogate marker in our trial design will likely neglect the potential benefit of these agents in delaying tumor progression. In this regard, the PCWG2 recommended the utilization of prevent/delay endpoints in these trials rather than control/relieve/eliminate endpoints commonly used for cytotoxic agents [29]. Thus, an alternative endpoint may be needed for this group of targeted agents.

Whether PFS is a justifiable intermediate surrogate endpoint in prostate cancer is still an open question. PFS is defined as the time from study entry or randomization of a patient until objective tumor progression or death. The use of PFS in trials has several advantages that include a smaller sample size and shorter duration of follow-up. PFS is also not affected by crossover or subsequent treatments. Various definitions for PFS, including PSA, radiographic changes, new metastatic lesions, and disease-related symptoms, have been used in clinical practice. However, data has shown that current measures of PFS for men with CRPC are not strong surrogates for OS [35,36]. It again was demonstrated by the failed phase III CALGB 90401 trial comparing docetaxel with or without bevacizumab in mCRPC with OS as the primary endpoint [37]. The development of this trial is based on favorable PFS of 8 months in bevacizumab arm in phase II CALGB 90006 trial [38]. Although improvements were seen again in PFS, the bevacizumab arm did not improve OS and was associated with greater morbidity and mortality in this large scale trial. In the phase III SPARC (Satraplatin and Prednisone Against Refractory Cancer) trial evaluating the use of satraplatin as second-line therapy in mCRPC, a composite PFS end point was utilized. It consisted of radiographic progression, symptomatic progression (pain, analgesics, ECOG performance status, weight loss, etc.), and occurrences of skeletal-related events [39]. Although the treatment of satraplatin plus prednisone was associated with a statistically significant improvement in the composite PFS endpoint (11.1 vs. 9.7 weeks; P < 0.0001), it did not translate into a meaningful clinical benefit such as a prolonged survival [39]. A meta-analysis of multiple trials, to verify potential intermediate surrogates, detected that PFS is an acceptable surrogate in advanced colorectal and ovarian cancer, but not in breast and prostate cancer [40]. It is unclear whether the discrepancy is due to hormonal modulation or the predominance of bone metastasis not assessable by RECIST in the majority of breast or prostate cancer cases. PFS appears to not be an optimal surrogate for OS in circumstances where disease is confined to bone. The current guideline only requires either “no new lesions” or “new lesions” being documented [29] for bone disease assessment and may have overlooked subtle changes in bone which carries meaningful survival value. The ability to accurately determine disease status in prostate cancer, i.e. PFS, is likely compromised given the lack of reproducible and effective modalities to delineate changes of bone metastases.

## Molecular phenotypes and biomarker development

Given that there are no validated surrogates of OS to assess early clinical benefit in CRPC trials, incorporation of relevant biomarkers in early phases of clinical development is essential to improve the success of late phase III testing. Clinical trial design is challenged by the ability to correlate clinical outcome with specific biomarkers reflective of the tumor phenotype with predictive or prognostic value. PSA has long been used as prognostic marker but due to its limitations, various PSA-based biomarkers were explored such as PSA velocity, PSA doubling time, etc. A systematic review of the literature highlighted conflicting data from heterogeneous studies on the use of such biomarkers as prognostic risk factors; [41] thus, the PCWG2 has discouraged the use of post-therapy changes of PSA doubling time as a primary endpoint [29].

However, resistance is bound to occur as the median duration of radiographic response is 5.6 months and 47 weeks, in patients treated with AA and MDV3100, respectively [14]. Acquired drug resistance has been a problem with many other targeted cancer agents. Finding strategies to overcome resistance is of paramount importance. If predictive marker(s) for androgen deprivation therapy (ADT) could be pinpointed to patients who are more likely to benefit, then unnecessary exposure of toxicity to non-responders would be avoided and the efficacy of these novel agents would be enhanced. Genetic biomarkers or fingerprints of mCRPC will yield specific information about the tumor and lead to a better understanding of the disease and the development of more effective treatment options. In addition, depending on the prior therapy received, metastatic tumors might acquire mutations or genetic changes that were clonally selected to confer a survival advantage. Obtaining tissue samples at various stages of the disease for verification of the evolving biology of prostate cancer progression may be particularly difficult owing to the heterogeneous and multifocal nature of the disease. Many studies have attempted to identify prostate cancer signature gene(s) and other genetic alterations, and new findings are emerging. A population-based study proved the prevalence of *ETS* fusions with *TMPRSS2* as the first recurrent genomic alteration in prostate cancer. The ETS-related gene (*ERG*) is the most common fusion partner for the androgen-regulated gene *TMPRSS2*[42]. The fact that *ETS* fusion-positive prostate cancer usually carries a more aggressive phenotype but still respond to ADT owing to its 5’ androgen-regulated partner, makes it an ideal potential predictive biomarker for ADT. In fact, increased prevalence of patients with hormone-regulated *ERG* gene rearrangement was identified in a group of mCRPC patients treated with AA who had more than 90% PSA declines [43]. However, in an analysis of *TMPRSS2-ERG fusion* status in patients enrolled in sequential phase II AA trials, similar rate of PSA response were indentified in both groups regardless of the presence of *TMPRSS2-ERG* fusion. Thus, the predictive value of *TMPRSS2-ERG* for the response to hormonal treatment is still uncertain at this time. Moreover, it has been technically challenging in developing inhibitors of *ETS* fusions targeting the 3’ *ETS* due to its poor accessibility [44]. Newer technology such as RNA interference might hold therapeutic promise and needs to be further explored [45]. Other than gene fusions, germline DNA polymorphisms may be associated with the response or resistance to ADT and increased uptake of testosterone in advanced prostate cancer [46]. If these genetic biomarkers can be validated in a large cohort of patients, they may be incorporated into future prospective clinical trials as both prognostic biomarkers to identify high-risk patients and predictive markers for patient stratification before initiating therapy.

Investigations are ongoing to develop more specific and sensitive tools to detect bone changes in prostate cancer. PET scans with different tracers have been studied with various success for its ability to detect metastatic diseases, monitor response to therapies and prognosticate OS [47]. Digital-contrast enhanced-magnetic resonance imaging was also employed. In a phase II trial using AZD2171, it exhibited correlation between targeted activity and tumor response [48]. Results from all these efforts will likely contribute to guideline updates of the criteria for evaluation of bone diseases in the near future.

Circulating tumor cell (CTC) count has also been widely implemented in various clinical trials and can be used for risk-stratification, molecular subclassification, or as a surrogate endpoint in treatment efficacy studies. The decline of CTC count after various treatments has been associated with improved OS [49,50]. The clinical utility of monitoring CTC changes with treatment is currently undergoing the efficacy-response biomarker qualification process, specifically as an intermediate endpoint for detecting survival benefit from AR-targeted therapies. The enumeration of CTC has been incorporated into several phase II studies including trials of AA [51-53] with additional surrogacy analyses embedded in phase III survival-based trials. While changes in CTCs have also been proposed as possible pharmacodynamic markers of immune-based therapies, such as sipuleucel-T or ipilimumab, their role remains to be determined. Molecular determinants can be identified and characterized in CTCs as potential predictive markers of drug sensitivity or resistance to treatment. There are a range of assessment methods that are in various stages of development, validation, and clinical testing. Presently, CellSearch (Veridex) is the only CTC assay that is FDA-cleared for use in the clinic. Further development of CTC evaluation technologies could facilitate the subclassification of patients based on molecular profiles or a CTC-derived molecular signature to allow for patient selection for targeted therapies or to guide treatment selection. Prospectively designed studies should further assess the role of CTC biomarkers as strong intermediate endpoints for clinical benefit, in determining prognosis and monitoring treatment effects to incorporate into clinical practice, and for accelerating drug development.

## Strategies for combination therapies

Several new treatment options have recently become available for patients with mCRPC. Clinicians are now faced with the challenge of understanding how to best utilize these newer therapies. The post-docetaxel era has seen numerous trials combining docetaxel with multiple agents with distinct mechanisms of action including TKIs, angiogenic inhibitors, bone-targeted agents, BCL-2 inhibitors, chemotherapies, immunologic agents, and vitamin D analogs [10]. Analysis on a phase II combination trial of sunitinib and docetaxel showed a 56% PSA response rate and 42% RECIST-defined response rate [54]. A phase III randomized trial with this combination is currently underway. However, two recent phase III trials combining docetaxel with an antiangiogenic agent, either bevacizumab (CALGB 90401) or lenalidomide (MAINSAIL), failed to improve OS in mCRPC patients [55,56]. Indeed, blocking neovascularization by VEGF antibody was found to induce hypoxia which in turn up-regulate angiogenic factors such as bFGF, chemokines and ephrin, and angiopoietin families, leading to anti-angiogenesis rescue [57]. To overcome the hurdle of multiple pre-existing or induced pro-angiogenic factors, a strategy combining docetaxel with dual antiangiogenic agents with distinct anti-angiogenic properties, bevacizumab and thalidomide, represented a new approach, and was demonstrated in a phase II trial. It resulted in high response rates in PSA changes and measurable diseases, and an encouraging estimated median survival rate in this patient population [58]. This demonstrated the feasibility and potential activity of combining antiangiogenic agents of different mechanisms of action with docetaxel. Currently, an ongoing Phase II trial is investigating the four-drug treatment regimen with thalidomide being replaced by lenalidomide, a derivative of thalidomide with more potent immunostimulatory and antiangiogenic activity but less toxicity (ClinicalTrials.gov NCT00942578).

Hormonal therapy combined with docetaxel was also tested in a phase I trial using ketoconazole and docetaxel. Although docetaxel was given weekly (since the trial was designed and implemented before the availability of the TAX 327 data), active tumor response was observed in 62% of patients with PSA response, and 28% with partial response in measurable disease [59]. This trial has suggested the combination of cytotoxic and hormonal agents might render an additive and possible synergistic antitumor activity. Several multicenter phase III trials combining first-line conventional hormonal agents with docetaxel are currently ongoing to address the question regarding early versus late initiation of chemotherapy in metastatic, hormonal-sensitive patients (ClinicalTrials.gov NCT00268476, NCT00104715). The potency of ketoconazole in controlling disease/PSA progression is usually not durable as adrenal androgen levels were increased relative to their nadir at the time of disease progression [60]. AA, on the other hand, has been shown to be able to continuously inhibit adrenal androgen synthesis even at the time of disease progression, as evident by continued suppressed level of serum androgen in several phase I/II studies of AA [51,61]. This observation excluded the possibility of resistance to AA due to mutated CYP17 and inefficient androgen blockade. It affirmed that the enzyme remained a valid target and still required sustained application even at the time of progression and initiation of subsequent therapies. Perhaps a combination therapy that integrates uninterrupted androgen deprivation by a novel, potent agent like AA and cytotoxic agents with a proven survival benefit such as docetaxel or cabazitaxel, will likely provide synergistic clinical benefit and overcome resistance in mCRPC patients. Such trials are currently underway in phase I testing (ClinicalTrials.gov NCT01400555, NCT01511536).

## Conclusions

In order to achieve sustained success in drug development, researchers must remain conscious of the challenges and limitations in conducting clinical trials in prostate cancer. Given the heterogeneity of the disease at the molecular level, it remains a challenge to identify suitable druggable target(s). Subsequent molecular studies are warranted to justify and/or validate the targeted therapy under investigation. Failures from several recent phase III trials based on expedited approaches in advancing a drug from phase I/II to III despite weak early phase trial data have taught us to be more selective about what agents to bring forward to be tested in the phase III setting. Reducing drug failures early in development is far more important than filling a pipeline with poorly chosen late-stage drugs.

Biomarkers can revolutionize both the development and use of targeted therapeutics, but is contingent upon the establishment of a concrete validation process. During the qualification process, a biomarker should be tested in phase I and II efficacy trials to measure its robustness and validate its association with predicted clinical outcome. The incorporation of mechanism-based biomarker endpoints into early clinical trials has facilitated in making early “go-no go” decisions in drug development [62,63] and reduces the risk of drug failures in large-scale, costly phase III trials. Finding reliable indicators of clinical efficacy of noncytotoxics or targeted agents is an area in need of further development. Existing intermediate surrogate biomarkers and easily accessible surrogates, such as CTC, can be analyzed and validated prospectively in large phase III trials and can be vital to the understanding of the benefits of particular agents and treatment combinations. Whether OS remains the gold standard or the reliability of PFS as a surrogate marker for emerging therapeutics will need to be further clarified in future studies.

## Appendix

**Metastatic castration resistant prostate cancer**: Progressive metastatic prostate cancer despite castrate level of testosterone.

**Surrogate endpoints**: Outcome measures that are intended to substitute for a clinical endpoint and are expected to predict clinical benefit based on scientific evidence.

**Promiscuous androgen receptor**: Androgen receptor protein that acquired mutations leading to the activation of the androgen-signaling axis with ligands other than testosterone and dihydrotestosterone.

**Circulating tumor cell count**: Enumeration of subpopulation of tumor cells derived from the primary cancer site which has been shown to correlate with survival in metastatic prostate cancer.

**Prostate cancer clinical trials working group**: A group of expert clinical investigators who meet periodically in an effort to standardize the eligibility and outcome measures in clinical trials in patients with metastatic castration resistant prostate cancer.

## Abbreviations

mCRPC, metastatic castration-resistant prostate cancer; AA, abiraterone acetate; AR, androgen receptor; TKI, tyrosine kinase inhibitor; OS, overall survival; PFS, progression-free survival; PSA, prostate-specific antigen; PCWG, prostate cancer clinical trials working group; ERG, ETS-related gene; CTC, circulating tumor cell.

## Competing interests

The authors indicated no potential conflicts of interests.

## Authors’ contributions

All authors participated in concept design, data collection and analysis, drafting and critically revising the manuscript. All authors read and approve the final manuscript.

## Authors’ information

Dr. William D. Figg is an internationally recognized pharmacologist and principle investigator of multiple prostate cancer clinical trials at the National Cancer Institute. He and his colleagues have made many original contributions in the advancement of prostate cancer therapeutics.
